# Auswirkungen von Lithium, Valproat, Carbamazepin und Antipsychotika auf die Kognition bei bipolarer Störung – ein systematisches Review

**DOI:** 10.1007/s00115-023-01454-y

**Published:** 2023-03-15

**Authors:** Sophie Leopold, Arnim Quante

**Affiliations:** 1 Abteilung für Psychotherapie und Psychiatrie, Charité – Universitätsmedizin Berlin, Campus Benjamin Franklin, Berlin, Deutschland; 2 Abteilung für Psychotherapie und Psychiatrie, Friedrich von Bodelschwingh-Klinik, Landhausstraße 33–35, 10717 Berlin, Deutschland

**Keywords:** Stimmungsstabilisatoren, Demenz, Pharmakologie, Hippocampus, Langzeitbehandlung, Mood stabilizers, Dementia, Pharmacology, Hippocampus, Long-term treatment

## Abstract

**Hintergrund:**

Die Langzeitauswirkungen der möglichen Therapieoptionen bei bipolarer Störung sind wenig untersucht, besonders in Bezug auf kognitive Beeinträchtigungen. Unterschiedliche Studien enthalten Hinweise, dass je nach Therapieform, das Risiko, an einer Demenz zu erkranken, weiter erhöht, aber auch gesenkt werden kann. Der aktuelle Forschungsstand wird in diesem systematischen Review zusammengefasst.

**Ziel der Arbeit:**

Die Auswirkungen einer Langzeittherapie mit Lithium, Valproat, Carbamazepin und Antipsychotika auf die Entstehung einer Demenz und kognitiver Beeinträchtigungen bei Patient*innen mit bipolarer Störung werden untersucht.

**Methoden:**

Es wurde eine systematische Literaturrecherche in der PubMed-Datenbank von den beiden Autor*innen im Zeitraum Mai bis Juli 2022 durchgeführt. Placebokontrollierte Studien, Metaanalysen, prospektive Studien mit Kontrollsubstanz, Fall-Kontroll-Studien, Kohortenstudien, systematische Reviews und randomisierte kontrollierte Studien wurden eingeschlossen.

**Ergebnisse:**

Der Großteil der verfügbaren Studien sieht in der Langzeittherapie mit Lithium einen protektiven Effekt auf das Gedächtnis und damit das Entstehen einer Demenz. Für Valproat hingegen wird ein eher negativer Einfluss beschrieben. Bei Antipsychotika ist die derzeitige Datenlage nicht aussagekräftig genug, aber auch hier wird ein eher neutraler bis negativer Einfluss mit der Langzeiteinnahme assoziiert.

**Diskussion:**

Lithium sollte, auch aufgrund des neuroprotektiven Effekts, in der Erhaltungstherapie der bipolaren Störung empfohlen werden. Der Einsatz von Valproat hingegen sollte in dieser Indikation kritisch gesehen werden. Antipsychotika sind bezüglich dieser Fragestellung noch nicht hinreichend untersucht, sodass keine generelle Empfehlung ausgesprochen werden kann.

## Abstract (en)

**Background:**

Until now the long-term consequences of the medical treatment for bipolar disorder have barely been examined, especially the consequences with respect to cognitive impairment and dementia. Some studies show signs that some treatment options have a better effect on the brain than others. This review summarizes the current state of research.

**Objective:**

The effects of long-term consequences of lithium, valproic acid, carbamazepine and antipsychotic agents on the development of dementia or cognitive impairments in patients with bipolar disorder were investigated.

**Methods:**

A systematic literature search was carried out in the PubMed data base from May to July 2022.

**Results:**

The majority of studies showed that lithium has a neuroprotective effect and can lower the risk of developing dementia, whereas an increased risk was found in patients taking valproic acid. There are only very few studies that deal with antipsychotic medication and the long-term consequences concerning dementia.

**Conclusion:**

Lithium should be recommended for the long-term treatment of bipolar disorder. Valproic acid should not or carefully be used as it can affect the risk of developing dementia. With respect to antipsychotics there is no recommendation as more studies are needed to evaluate the long-term consequences.

## Einleitung

### Überblick über die bipolare Störung

Die bipolare Störung (BS) ist charakterisiert durch mindestens zwei Episoden manischer oder hypomanischer und depressiver Episoden [6, 13]. Die Lebenszeitprävalenz der BS beträgt in Deutschland etwa 3 % [6].

Patient*innen mit BS haben im Verlauf ein erhöhtes Risiko für neurodegenerative Erkrankungen. Insbesondere das Risiko, an Demenz zu erkranken, kann durch die BS erhöht sein [1, 2, 8, 22]. Da auch die Alzheimer-Erkrankung, die häufigste Demenzform, in der Regel mit einer neuronalen Atrophie des Hippocampus einhergeht, könnte das in Studien nachweislich verringerte Volumen bei Patient*innen mit BS ebenfalls mit einem erhöhten Risiko für eine Alzheimer-Demenz assoziiert sein [11, 26].

### Pharmakologische Langzeittherapie der bipolaren Störung

Patient*innen mit einer BS müssen meistens lebenslang pharmakologisch behandelt werden.

Die effektivste medikamentöse Phasenprophylaxe ist die Behandlung mit Lithium [6]. Auch Antikonvulsiva wie Valproat oder Carbamazepin werden bei der BS eingesetzt [12].

Studien zeigen, dass Lithium das Risiko für kognitive Störungen reduzieren kann [29]. Die Behandlung mit Valproat hingegen kann das Risiko einer Demenz um bis zu 95 % steigern [28].

Zur Therapie der Manie und bei Ansprechen auch zur Phasenprophylaxe sind außerdem Antipsychotika (AP) wie Olanzapin, Arpiprazol oder Risperidon zugelassen sowie Quetiapin für alle Phasen der BS [6]. Bei den AP gibt es bei der Langzeitbehandlung von Schizophrenien ebenfalls Hinweise, dass sie einen negativen Effekt auf die kognitive Funktion haben können [27]. Die Studienlage zu AP und auch Carbamazepin in Bezug auf Risiken für Demenzerkrankungen ist dünn.

### Ziel der Arbeit

In diesem systematischen Review wird das Risiko für Demenzerkrankungen unter Lithium, Valproat, Carbamazepin und AP bei Patient*innen mit BS untersucht.

## Methoden

### Inklusions- und Exklusionskriterien

Eingeschlossen wurden placebokontrollierte Studien, Metaanalysen, prospektive Studien mit Kontrollsubstanz, Fall-Kontroll-Studien, Kohortenstudien, systematische Reviews und randomisierte kontrollierte Studien, die sich mit der Langzeittherapie bzw. Phasenprophylaxe mit Lithium, Valproat, Carbamazepin und AP und deren Auswirkung auf die kognitive Funktion bzw. das Entstehen einer Demenz befassen. Zudem wurden Studien mit Fokus auf Volumenänderungen des Hippocampus unter Pharmakotherapie bei BS eingeschlossen. Ausgeschlossen wurden tierexperimentelle Studien, Studien mit Fokus auf andere psychiatrische Erkrankungen oder bei Gesunden sowie Studien mit abweichenden pharmakotherapeutischen Strategien. Pharmakotherapien, die nur für eine Episode der BS oder nur in Kombination mit anderen Pharmaka eingesetzt werden, wurden nicht eingeschlossen. Studien mit einer Therapiedauer von unter 28 Tagen wurden ebenfalls ausgeschlossen.

### Suchstrategie

Es wurde eine systematische Literaturrecherche von zwei Autor*innen in der elektronischen PubMed-Datenbank von Mai bis Juli 2022 durchgeführt. Für die Suche wurden die Termini „bipolar disorder“ „dementia“ „cognitive functioning“ „lithium“ „valproic acid“ „antipsychotics“ „carbamazepine“ „hippocampal volume“ „hippocampus“ verwendet.

**Figure 1:**
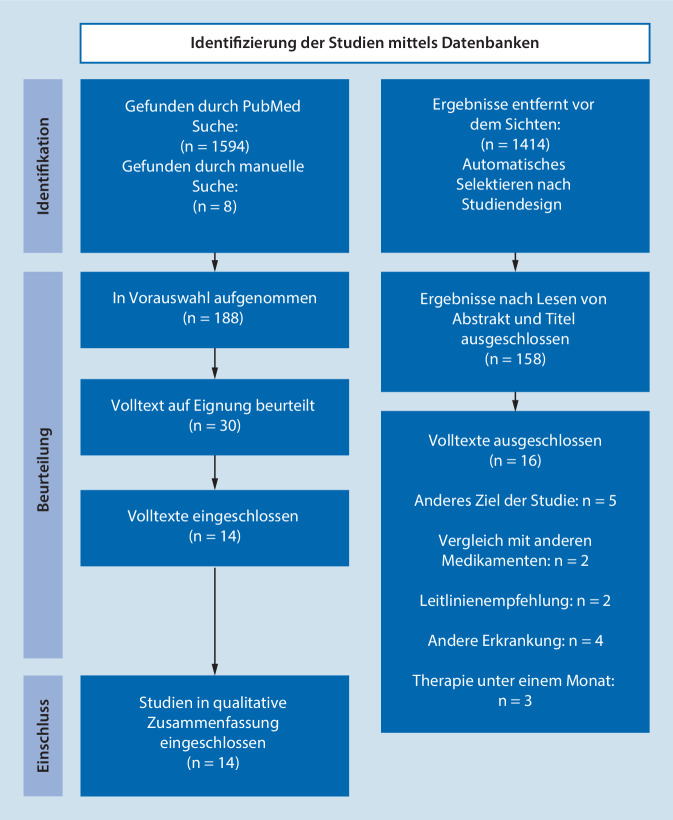

Die initialen Suchergebnisse wurden nach Studiendesign gefiltert und durch das Lesen von Abstrakt und Titel entweder ein- oder ausgeschlossen. Anschließend wurden die verbleibenden Volltexte auf Eignung beurteilt (Abb. 1).

### Qualitätsbeurteilung

Fall-Kontroll- und Kohortenstudien wurden anhand der Newcastle-Ottawa Scale beurteilt [30]. Randomisierte kontrollierte Studien wurden mithilfe des Jadad-Scores nach Jadad et al. qualitativ bewertet [14].

## Ergebnisse

### Überblick über die Ergebnisse

Die initiale PubMed-Recherche ergab 1594 Treffer, von denen 1414 nicht dem eingeschlossenen Studiendesign entsprachen. Nach dem manuellen Sichten der systematischen Reviews wurden 8 weitere Studien identifiziert und in die Auswahl aufgenommen. Durch das Sichten von Titel und Abstrakt verblieben 30 Volltexte. Die Volltexte wurden gelesen und 16 Studien ausgeschlossen.

Insgesamt ergab die Suche 14 Ergebnisse, die in dieses systematische Review inkludiert wurden. Tab. 1 listet alle inkludierten Studien auf.

**Table 1:** 

Referenz	Population/Einschlusskriterien	Behandlung + Kontrollgruppe	Durchschnittliche Dosierung	Fallzahl	Ergebnis	Studiendesign und -dauer	Qualität (Jadad oder Newcastle-Otawa-Skala)
Daban et al. (2006) [4]	Pat. mit BS Typ I und II	1) Lamotrigin	k. A.	1) 15	Lamotrigin hat keinen negativen Effekt auf das Gedächtnis	Fall-Kontroll-Studie	7/9
		2) Carbamazepin/Valproat		2) 18			
Daglas et al. (2016) [5]	Pat. mit BS Typ I nach erster manischer Phase zwischen 15 und 25 Jahren	1) Lithium	1,0–0,6	1) 17	Signifikanter Unterschied bezogen auf flüssige Sprache, sonst keine signifikanten Unterschiede	Randomisierte kontrollierte Studie, 12 Monate	4/5
		2) Quetiapin		2) 17			
Gerhard et al. (2015) [9]	Pat. mit BS-Typ I und II > 50 Jahre	1) Lithium	k. A.	1) 6900	Signifikanter positiver Effekt von Lithium auf Demenz	Retrospektive Kohortenstudien, 01.01.2001 bis 31.12.2004	8/9
		2) Neg. Kontrollgruppe: Antikonvulsiva		2) 20.778			
		3) Pat. ohne Behandlung		3) 18.119			
Hajek et al. (2012) [10]	Pat. mit BS Typ I und II	1) Lithium	1) zwischen 600–2100 mg/Tag	1) 101	Hippocampus größer bei Pat. mit Lithiumbehandlung	Metaanalyse	k. A.
		2) Kein Lithium	–	2) 245			
		3) Gesunde Kontrollgruppe	–	3) 456			
Haukvik et al. (2022) [11]	Pat. mit BS Typ I und II	1) Lithium	k. A.	1) 319	Kein Unterschied zwischen Lithium und gesunder Kontrolle bei Hippocampusvolumen, Gruppe 3 im Vergleich kleinere Hippocampi	Metaanalyse	k. A.
		2) Gesunde Kontrolle		2) 3226			
		3) Antipsychotika/Antikonvulsiva		3) 464			
Mora et al. (2016) [18]	Euthyme Pat. mit BS	1) Lithium	k. A.	1) 10	Signifikante kognitive Einschränkung bei Pat. mit BS	Kohortenstudie, 6 Jahre	7/9
		2) Gesunde Kontrollgruppe		2) 10			
Muralidharan et al. (2015) [19]	Pat. mit BS Typ I	1) Lithium	1) Durchschnittlich 957 mg/Tag	1) 34	Therapie mit Valproat negativeren Effekt auf Arbeitsgedächtnis als Lithium	Fall-Kontroll-Studie, 3 Monate	8/9
		2) Valproat	2) Durchschnittlich 1005 mg/Tag	2) 38			
		3) Gesunde Kontrollgruppe	–	3) 40			
Pigoni et al. (2020) [20]	Pat. mit BS	1) Valproat	1) 1005–1250 mg/Tag	–	Valproat eher negative Auswirkungen, insbesondere auf das Arbeitsgedächtnis	Minireview	k. A.
		2) Gesunde Kontrollgruppe	–				
Saito et al. (2017) [23]	Japanische Bürger*innen mit BS Typ I und II	1) Lithium	k. A.	1) 96	Therapie mit Lithium hat keinen Effekt auf Kognition	Interventionsstudie, keine Dauer	5/9
		2) Gesunde Kontrollgruppe		2) 196			
Senturk et al. (2007) [25]	Pat. mit BS Typ I und II	1) Lithium	1) 600–1800 mg/Tag	1) 17	Verbales Gedächtnis in Gruppe 1 und 2 eingeschränkt	Fall-Kontroll-Studie	6/9
		2) Valproat	2) 500–2000 mg/Tag	2) 11			
		3) Gesunde Kontrollgruppe	–	3) 29			
Torrent et al. (2011) [27]	Pat. mit BS Typ I und II zwischen 18 und 65 Jahren	1) Quetiapin	1) 404,1 mg/Tag	1) 12	In semantischer Wortflüssigkeit Gruppe 1–3 schlechter als Gruppe 4, Gruppe 2 in Erkennungsaufgaben schlechter	Fall-Kontroll-Studie	9/9
		2) Olanzapin	2) 7,7 mg/Tag	2) 26			
		3) Risperidon	3) 3,7 mg/Tag	3) 30			
		4) Kein Medikament	–	4) 16			
		5) Gesunde Kontrollgruppe	–	5) 35			
Tsai et al. (2016) [28]	Pat. aus Taiwan mit BS Typ I und II	1) Valproat	k. A.	1) 1792	Signifikant erhöhtes Risiko für Demenz bei Behandlung mit Valproat	Kohortenstudie, 3 Jahre	7/9
		2) Kein Valproat		2) 3366			
Velosa et al. (2020) [29]	Pat. mit BS	1) Lithium	k. A.	1) 6483	Lithium senkt Risiko für Demenz um 49 %	Metaanalyse, 3–17 Jahre	k. A.
		2) Kein Lithium		2) 43.496			
Yucel et al. (2007) [31]	Pat. mit BS Typ I und II	1) Lithium	Serumlevel: 0,5–0,7	1) 12	Hippocampusvolumen nimmt bei Lithiumtherapie zu	Longitudinalstudie, 2–4 Jahre	k. A.

*k.* *A.* keine Angabe, *BS* bipolare Störung, *Pat.* Patient*innen

### Lithium

#### Auswirkung auf das Risiko einer Demenz

Zwei Studien untersuchen das direkte Risiko von Lithium auf die Entstehung einer Demenz. Die retrospektive Kohortenstudie von Gerhard et al. mit einer hohen Fallzahl von *n* = 27.678 Patient*innen mit BS und einer Kontrollgruppe von *n* = 18.119 konnte einen statistisch signifikanten, positiven Effekt von Lithium, sofern es über mindestens 300 Tage eingenommen wurde, feststellen (Hazard Ratio [HR] = 0,77, 95 %-CI 0,60–0,99; [9]). Auch in der Metaanalyse von Velosa et al., in der auch die Studie von Gerhard et al. eingeschlossen wurde, zeigte sich eine Risikoreduktion um 49 % für Demenzen unter Lithium [29]. Auch hier war die Fallzahl mit *n* = 49.979 eher hoch.

#### Auswirkung auf kognitive Beeinträchtigungen

Insgesamt 5 Studien untersuchen den Einfluss von Lithium auf die kognitive Funktion. Mora et al. untersuchte 10 Patient*innen mit BS, die auf die Therapie mit Lithium angesprochen haben im Vergleich zu 10 gesunden Kontrollen. Auch wenn Patient*innen unter Lithium in den kognitiven Bereichen schlechter abschnitten – mutmaßlich aufgrund der BS an sich – sehen Mora et al. einen Erfolg darin, dass sich die kognitive Leistung nach 6 Jahren unter der Therapie nicht verschlechterte [18].

In der Studie von Saito et al. wurden mittels des Alda-Scores (The Retrospective Assessment of the Lithium Response Phenotype; [24]) und des BACS-Scores (The Brief Assessment of Cognition in Schizophrenia; [15]) das Ansprechen von Lithium und Auswirkungen auf die Kognition untersucht. Es konnte keine Verbindung zwischen Ansprechen und Kognition festgestellt werden, wodurch die Autoren zu der Schlussfolgerung kommen, dass Lithium keinen Effekt auf die Kognition habe. Da der BACS-Score allerdings nur für Patient*innen mit Schizophrenie evaluiert ist, sollten die Studienergebnisse mit Vorsicht interpretiert werden [23].

Zwei Studien verglichen die Effekte von Lithium und Valproat auf kognitive Leistungen bei BS im Vergleich zu gesunden Kontrollen. In einer dieser Studien führte die Behandlung mit Lithium im Vergleich zu Valproat zu keiner Verschlechterung des Arbeitsgedächtnisses (*p* < 0,001). In allen anderen untersuchten Bereichen wiesen die Gruppen keinen signifikanten Unterschied auf (*p* > 0,13; [19]). Im Gegensatz dazu zeigte sich in der Studie von Senturk et al. eine signifikante Verschlechterung des verbalen Gedächtnisses unter Lithium oder Valproat im Vergleich zu einer Kontrollgruppe (*p* = 0,018; [25]).

Daglas et al. führten eine randomisierte kontrollierte Studie durch, in welcher sie die kognitive Funktion unter der Behandlung mit Lithium oder Quetiapin nach 3 und 12 Monaten verglichen. Die Lithiumgruppe wies eine signifikante Besserung der Wortflüssigkeit im Vergleich von 3 zu 12 Monaten auf (*p* = 0,004). Bei Quetiapin konnte dieser Effekt nicht nachgewiesen werden. Dafür nahm die Fehlerrate bei Aufgaben der Problemlösung in der Lithiumgruppe zu (*p* = 0,045), in der Quetiapingruppe hingegen nicht [5].

#### Auswirkungen auf das Hippocampusvolumen

In einer Metaanalyse konnte festgestellt werden, dass das Hippocampusvolumen im Langzeitverlauf der BS schrumpft [11]. Der Einfluss von Lithium auf das Hippocampusvolumen wurde in 3 Studien untersucht. In der Metaanalyse von Haukvik et al. wurde Lithium mit AP oder Antikonvulsiva sowie gesunden Kontrollgruppen analysiert, in der Metaanalyse von Hajek et al. wurde Lithium vs. Kontrollgruppe bezüglich der Effekte auf den Hippocampus untersucht. In beiden Studien zeigte Lithium einen hypertrophierenden Einfluss auf das Hippocampusvolumen, während die Behandlung mit AP oder Antikonvulsiva mit einem signifikant kleineren Hippocampusvolumen einherging [10, 11]. In der Beobachtungsstudie von Yucel et al. konnte ebenfalls eine Zunahme des Hippocampusvolumens unter Lithiumtherapie nachgewiesen werden [31].

### Valproat

#### Auswirkungen auf das Risiko einer Demenz

Die Kohortenstudie von Tsai et al. untersuchte das Risiko für die Entwicklung demenzieller Erkrankungen unter Valproat. Es konnte gezeigt werden, dass das Risiko für die Entwicklung einer Demenz unter Valproat um 73–95 % erhöht ist (*p* = 0,001; [28]). Die Fallzahl der Patient*innen ohne Valproatbehandlung ist hier allerdings 3‑mal höher als die mit einer Valproattherapie. In der bereits oben beschriebenen Metaanalyse von Gerhard et al. zeigte sich, dass sämtliche Antikonvulsiva weder mit einem erhöhten noch mit einem reduzierten Risiko für die Entwicklung von Demenzen assoziiert waren [9].

#### Auswirkung auf kognitive Beeinträchtigungen

Drei Studien untersuchen den Effekt von Valproat auf die kognitive Funktion. In 2 Studien konnte ein negativer Effekt nachgewiesen werden [19, 20]. So konnte in einer Studie festgestellt werden, dass die Leistungen des Arbeitsgedächtnisses im Vergleich zu Patient*innen, die mit Lithium behandelt wurden, signifikant reduziert waren (*p* < 0,001; [19]). Dies wurde in dem Minireview von Pigoni et al., in welchem unter anderem die vorherige Studie eingeschlossen wurde, bestätigt [20]. In einer Studie von Daban et al., in der Lamotrigin mit Valproat oder Carbamazepin hinsichtlich kognitiver Störungen untersucht wurden, zeigte sich, dass Valproat und Carbamazepin mit einem schlechteren Ergebnis im Bereich Wortflüssigkeit assoziiert sind als Lamotrigin (*p* < 0,008; [4]).

#### Auswirkungen auf das Hippocampusvolumen

Mit Ausnahme der bereits oben erwähnten Metaanalyse zu Lithium und Antikonvulsiva konnten keine weiteren Studien zum Hippocampusvolumen unter Valproat identifiziert werden. Insbesondere der Hippocampusschwanz war unter Antikonvulsiva signifikant verkleinert [11].

### Antipsychotika

Es konnten keine Studien identifiziert werden, die das Risiko einer Langzeittherapie bei BS mit AP für Demenzerkrankungen untersuchen.

#### Auswirkungen auf kognitive Beeinträchtigungen

Auch konnten nur wenige Studien zu kognitiven Störungen gefunden werden. In der randomisierten kontrollierten Studie von Daglas et al. wurde die Beeinflussung von Quetiapin auf die Kognition bei jungen Patient*innen über einen Untersuchungszeitraum von 12 Monaten, untersucht. In dieser Studie konnten weder positive noch negative Auswirkungen auf die Kognition festgestellt werden [5].

Torrent et al. führten eine Fall-Kontroll-Studie durch, in der sie die Behandlung mit drei verschiedenen AP – Olanzapin, Risperidon und Quetiapin – mit unbehandelten Patient*innen und einer gesunden Kontrollgruppe verglichen. Patient*innen mit BS schnitten generell schlechter als die Kontrollgruppe ab. Olanzapin und Risperidon waren mit einer schlechteren verbalen Lernleistung assoziiert, während dieser negative Effekt bei Quetiapin nicht nachgewiesen werden konnte. Auf der anderen Seite waren Olanzapin und Risperidon mit besseren Ergebnissen im Trail-Making-Test assoziiert [27].

#### Auswirkungen auf das Hippocampusvolumen

Haukvik et al. identifizierte mit der bereits oben erwähnten Metaanalyse, dass AP mit einem geringerem Hippocampusvolumen assoziiert waren im Vergleich zu Lithium [11].

### Carbamazepin

Es konnten keine Studien zur Beurteilung des Risikos für die Entwicklung demenzieller Erkrankungen oder kognitiver Störungen unter Langzeittherapie mit Carbamazepin bei BS identifiziert werden. Lediglich in der Studie von Gerhard et al. konnte für sämtliche Stimmungsstabilisatoren weder ein reduziertes noch erhöhtes Risiko beschrieben werden [9].

## Diskussion

### Hauptergebnisse

Verschiedene medikamentöse Therapiestrategien in der Behandlung der BS können unterschiedliche Auswirkungen auf die Kognition und auf das Risiko für die Entwicklung demenzieller Erkrankungen haben. Da die BS per se mit einem erhöhten Risiko für kognitive Störungen einhergeht und sich das Risiko mit zunehmender Anzahl an Episoden weiter erhöhen kann, ist eine Differenzierung der Genese der kognitiven Defizite nicht immer möglich. Um das Risiko zu minimieren, sollte die Pharmakotherapie der BS auch unter diesem Aspekt, insbesondere in der Langzeittherapie, ausgewählt werden. Für Lithium könnte, anhand der vorhandenen Studien, ein neutraler, teilweise aber auch ein protektiver Einfluss auf kognitive Defizite und die Entwicklung demenzieller Erkrankungen vorteilhaft bei der Therapie sein [9, 29]. Anhand der Studien für Valproat lässt sich ableiten, dass Valproat mit einem erhöhten Risiko für demenzielle Erkrankungen einhergehen könnte. Es zeigten sich eher negative Auswirkungen auf die Kognition: So konnten Hinweise auf kognitive Defizite sowohl bei einer kurzzeitigen als auch bei einer Langzeittherapie gefunden werden. In einer Studie zeigte sich in der Langzeittherapie ein signifikant erhöhtes Risiko für die Entstehung einer Demenz [9, 19, 20, 25, 28]. Jedoch müssten diese Aussagen mit weiteren Langzeitstudien reevaluiert werden.

Antipsychotika werden zunehmend in der Behandlung, teilweise in sämtlichen Phasen der BS eingesetzt. Umso erstaunlicher ist es, dass die Datenlage für AP im Hinblick auf kognitive Defizite sowohl bei der BS als auch bei anderen Erkrankungen sehr schwach ist. Während eine Studie Hinweise für kognitive Beeinträchtigungen enthielt, konnte eine andere Studie keine relevanten Einflüsse auf die Kognition nachweisen [5, 27]. Studien, die das Risiko für die Entstehung demenzieller Erkrankungen durch AP bei der Behandlung von BS untersuchen, konnten nicht identifiziert werden. Somit kann derzeit keine eindeutige Aussage zu AP bezüglich des Risikos für kognitive Störungen getroffen werden.

Studien zu Carbamazepin erfüllten nicht die Kriterien für dieses Review, jedoch finden sich in der Literatur Hinweise, dass auch Carbamazepin mit kognitiven Defiziten assoziiert sein könnte. Dias et al. beschreiben vor allem negative Effekte auf das Gedächtnis und die Aufmerksamkeit [7].

Im Hinblick auf das Hippocampusvolumen zeigte sich, dass Lithium einen protektiven Effekt zu haben scheint [10, 11, 31]. Im Vergleich dazu konnte in einer anderen Studie gezeigt werden, dass Antikonvulsiva und AP eher einen negativen Einfluss auf die Volumina zu haben scheinen [11]. Ob dieser Einfluss tatsächlich auf die Pharmaka zurückzuführen ist, bleibt jedoch unklar. Aufgrund der Datenlage ist eine Assoziierung zwischen dem Hippocampusvolumen und kognitiven Störungen bzw. dem Entstehen einer (Alzheimer‑)Demenz nicht eindeutig herstellbar, es kann jedoch als potenzielle Risikoerhöhung in Betracht gezogen werden.

Da die Qualität der hier eingeschlossenen Studien deutlich variiert und Informationen zur Dauer und Dosis der Einnahme, der Episodenhäufigkeit, zu wahnhaften Symptomen und Subtanzmissbrauch nicht immer vorhanden waren bzw. nur in einigen Studien aufgeführt wurden, könnten diese die Ergebnisse bezüglich eines erhöhten Risikos für demenzielle Erkrankungen verfälscht sein. Auch geschlechtsspezifische Unterschiede waren in den eingeschlossenen Studien häufig nicht zu finden. Daher sollten unsere Schlussfolgerungen mit Vorsicht interpretiert werden.

### Limitationen der Arbeit

Insgesamt ist die Studienlage zu diesem Thema schwach, sodass keine eindeutigen Aussagen getroffen werden können und die Ergebnisse eher als Hinweise zu verstehen sind. Da viele Studien nur eine geringe Probandenzahl aufweisen, ist die Aussagefähigkeit nochmals eingeschränkt. Dazu kommt, dass die Qualität der Studien teilweise sehr variierte; so konnte nur eine randomisierte kontrollierte Studie eingeschlossen werden, Studien mit Placebokontrolle oder mit Vergleichssubstanz waren kaum vorhanden.

Zudem wurden nur Studien aus der PubMed-Datenbank eingeschlossen, wodurch möglicherweise weitere Studien unberücksichtigt blieben.

Zu Carbamazepin konnten keine Studien eingeschlossen werden, weswegen dazu keine Aussage gemacht werden kann. Allerdings verliert Carbamazepin aufgrund der Interaktionen zunehmend an Bedeutung. Medikamente wie Lamotrigin oder Antidepressiva wurden gar nicht inkludiert, da diese in der Monotherapie der BS nicht zugelassen sind und somit auch keine validen Aussagen dieser Medikamente zum Demenzrisiko bei der BS gemacht werden können.

Der von uns gewählte Cut-off von 28 Tagen als Mindestdauer für eine Behandlung ist natürlich sehr kurz. Es ist anzunehmen, dass Langzeitstudien bestenfalls über Jahre deutlich aussagekräftiger wären. Auf der anderen Seite ist es anhand der Studienlage nicht eindeutig, ob auch kürzere Einnahmezeiträume nicht auch mit längerfristigen kognitiven Störungen assoziiert sein können.

### Schlussfolgerung

Bei der Behandlung der BS sollten auch Langzeitfolgen in den Entscheidungsprozess der Pharmakotherapie einfließen. Im Hinblick auf das Risiko für demenzielle Erkrankungen scheint Lithium im Vergleich zu Valproat vorteilhaft zu sein. Der Einsatz von Lithium bei leichten kognitiven Störungen oder der Alzheimer-Demenz wird derzeit erforscht und diskutiert [16]. Moon et al. diskutieren in ihrer Studie, dass gerade ältere Menschen mit BS eher mit Lithium behandelt werden sollten, um das Risiko für die Entstehung einer Demenz zu reduzieren [17].

Auch Chen et al. kommen in ihrer retrospektiven Kohortenstudie zu dem Ergebnis, dass eine Lithiumtherapie mit einer Reduktion des Demenzrisikos assoziiert sein kann, und bekräftigen damit die Aussage von Moon et al. [3].

Der Einsatz von Valproat scheint das Risiko für kognitive Defizite und demenzielle Erkrankungen zu erhöhen, weswegen eine Langzeittherapie als Phasenprophylaxe zumindest kritisch zu beurteilen ist. Dies ist auch bei der Therapie von anderen Erkrankungen wie beispielsweise der Epilepsie zu beobachten, weswegen die dauerhafte Behandlung mit Valproat sorgfältig abgewogen werden sollte [21].

Aufgrund der erstaunlich schwachen Datenlage zu AP kann keine Aussage zum Demenzrisiko getroffen werden. Durch die stetige Zunahme des Einsatzes von AP bei BS sollte die Auswirkung einer Langzeittherapie intensiver untersucht werden.

## Fazit für die Praxis

In diesem systematischen Review zeigte sich Lithium gegenüber Valproat in der Rückfallprophylaxe bei BS hinsichtlich der Entwicklung kognitiver Defizite überlegen. Erste Daten zeigen zudem, dass Lithium einen protektiven Effekt bez. der Entwicklung demenzieller Erkrankungen haben könnte. Dies sollte bei der Auswahl der Phasenprophylaxe unbedingt beachtet werden, Lithium also insbesondere dem Valproat vorgezogen werden. Auch sollte ein Umstellen für die Phasenprophylaxe erwogen werden, wenn Valproat, Antipsychotika oder Carbamazepin in der Akutbehandlung zum Einsatz gekommen sind. Leider ist die Datenlage von Antipsychotika und Carbamazepin zu schwach, um konkretere Handlungsempfehlungen auszusprechen.
